# Identifying effective candidates for sacro-iliac joint fusion

**DOI:** 10.2340/17453674.2024.41306

**Published:** 2024-08-21

**Authors:** Paul Gerdhem, Thomas Kibsgård, Engelke Randers

**Affiliations:** 1 Department of Surgical Sciences, Uppsala University, Sweden; 2 Department of Hand Surgery and Orthopaedics, Uppsala University Hospital, Sweden; 3 Division of Orthopaedic Surgery, Oslo University Hospital, Oslo, Norway; 4 Institute of Clinical Medicine, University of Oslo, Oslo, Norway

*Sir,–*We thank Daisuke Kurosawa and Bengt Sturesson for their interest in our paper [1]. In their letter we identified 6 comments that need to be answered, and for clarity we have abbreviated and rephrased them below.

1. 2nd paragraph: *Patients may improve after SI joint surgery, and especially if treated with lumbar fusion before SI joint surgery.* There is very little literature examining prognostic factors for outcomes after minimally invasive sacro-iliac joint fusion. To our knowledge only 2 articles have evaluated the effect of prior lumbar fusion on the outcome of minimally invasive sacro-iliac joint fusion, and neither could find that those with prior lumbar fusion had better outcomes [2,3].
  In our cohort study from the Swespine registry we excluded previously surgically treated patients to avoid difficulties in interpreting the outcome when patients had a history of previous surgery [4], but they were included in our recent placebo-controlled randomized trial (RCT) [4].
2. 3rd paragraph: *Females have a worse outcome.* That the vast majority were women in our study from the Swedish Spine register and previous publications illustrate that females more often than men have pain that seems to emanate from the SI joint [4-7]. Previous publications do not support that women have a worse postoperative outcome after sacro-iliac joint fusion than men [8].
3. 4th paragraph: *Post-traumatic patients have a better outcome.* Post-traumatic patients were not excluded in our randomized controlled trial [5], as long as they had been treated non-operatively and the fracture occured more than a year before inclusion, similar to other studies [6,7]. We have not found any reports which show that post-traumatic patients have a better outcome.
4. 5th paragraph: *Diagnostic procedures may differ in the different centers.* According to personal contacts with all centers, diagnostic procedures were similar.
5. 5th paragraph: *Missing data in one of the centers.* That the center treating the largest number of cases does not distribute preoperative Swespine questionnaires to patients is disappointing. In an elective setting this is easy to do, and patients are generally willing to complete such questionnaires. A larger number of patients included in our study would have strengthened the conclusions but not necessarily change them [9,10].
6. 6th paragraph: *Evaluating external fixation as a means to identify patients with SI joint pain should be performed.* We agree that methods to improve the selection of patients for surgery is important, irrespective of suspected disorder. However, external fixation has not been shown to be a successful addition in determining candidates for low back pain surgery when tested against placebo [11], and, as far as we know, reports on patients with SI joint pain are scarce and not controlled [12].

To conclude, based on 2 studies, 1 placebo-controlled surgical trial and 1 registry-based study with similar results [4,5], we question the use of SI joint fusion in the treatment of pelvic pain, and urge the medical community to continue to improve both diagnostics and treatment for women and men with disabling pelvic pain.
